# Tuberculosis

**Published:** 1892-12

**Authors:** 


### TUBERCULOSIS.

We too frequently hear exaggerated statements of the prevalence of tuberculosis, and the public are informed by seeming authorities and through the newspapers that large percentages of cattle through the country are affected with this disease. Certain veterinarians, after spending a few hours in a laboratory in conversation with some State health officer, are taken to a byre where a lot of cows are being milked to death, or to the shambles where a lot of steers show the result of a few months over-feeding with swill, and upon finding a number of diseased animals, become sanitary enthusiasts and fly to the public press to make a mountain out of a mole hill, demand impossible "scientific" (?) control of the cattle industry, and destroy a good herd of cattle for some good-natured philanthropist, or ruin the business of a few small farmers in the neighborhood.

Under the law which is printed below, we are glad to report some excellent and judicious work being done by Thomas Newbold, Esq., of the New York State Board of Health. He has organized a complete inspection of *all* of the cattle in an extensive agricultural district, under the guidance of Dr. Austin Peters, of Boston, and Dr. Cooper Curtice, of Moravia, N. Y. We shall shortly be able to give our readers a detailed and accurate account of these investigations and their results.

CHAPTER 487.—AN ACT IN RELATION TO TUBERCULOSIS IN MILCH COWS AND OTHER CATTLE AND INFECTIOUS AND CONTAGIOUS DISEASES OF CATTLE. APPROVED BY THE GOVERNOR MAY 5, 1892. PASSED, THREE-FIFTHS BEING PRESENT.

*The People of the State of New York, represented in Senate and Assembly, do enact as follows:*

SECTION 1. That the State Board of Health shall use all reasonable means for ascertaining the existence and cause of disease in, or danger to health from, milch cows and other cattle, in the State of New York, and for averting the same, and preventing all injury from tuberculosis in milch cows, or other cattle, in any part of the State; and shall promptly cause all proper information in possession of said Board of Health respecting any such disease among cattle in any part of the State to be sent to the local Board of Health of the city, village or town nearest to the herd, cows or other cattle affected by any such disease, and shall add thereto such useful suggestions as to the removal of all sources of danger therefrom or the destruction of said cattle, as the experience of the said Board may at any time supply. And it is made the duty of the health authorities throughout the State to supply the like information and suggestions to the State Board of Health respecting the existence of any infectious or contagious disease in milch cows or other cattle in any part of the State.

SEC. 2. The State Board of Health shall have power to employ such and so many medical and veterinary practitioners and other persons as it may, from time to time, deem necessary to assist in the inspection, in the isolation, destruction or disposition of milch cows or other cattle affected with tuberculosis or any infectious or contagious disease in any portion of the State; to prescribe rules and regulations for such inspectors and employes and to fix their compensation.

SEC. 3. Whenever tuberculosis shall be found among milch cows, or other cattle, in any part of the State, it shall be the duty of the State Board of Health to take measures to suppress said disease promptly, to prevent the same from spreading, and the said State Board of Health shall have the authority to order all persons to take such precautions against the spread of such disease as in the judgment of the said State Board of Health may be necessary or expedient; and, further, to call upon all sheriffs, deputy sheriffs, and officers of the peace in the neighborhood where such disease extends among milch cows or other cattle, to carry out and enforce the orders of said Board of Health respecting the same, and to observe and carry out the rules and orders and instructions which they may receive from the said Board of Health in the premises; and, further, to prescribe regulations for the destruction of animals affected with tuberculosis, or any contagious or infectious disease, and for the proper disposition of their hides and carcasses, and of all objects which might convey infection or contagion; provided, that no animal shall be destroyed unless first examined by medical or veterinary practitioner in the employ of the State Board of Health aforesaid; and, further, to prescribe rules and regulations for the disinfection of premises, buildings, boats, railroad cars, stables, and all objects from or by which infection or contagion may take place or be conveyed, and to alter and modify, from time to time, all such rules, regulations, orders and instructions as the said State Board of Health may deem expedient.

SEC. 4. Whenever in the judgment of the said State Board of Health it may become necessary to prevent the spread of tuberculosis or any infectious or contagious disease of domestic animals, or the public welfare shall be promoted thereby, the said

Board of Health may cause to be slaughtered, and has authority to kill, or order to be killed, any animal or animals which by contact or cohabitation with diseased animals, or by exposure to infection or contagion therefrom, the said State Board of Health may consider and determine, may be liable to contract or communicate the disease sought to be suppressed, and to prevent all danger therefrom.

SEC. 5. Any person or persons refusing to obey the orders, rules or regulations of the said Board of Health adopted in pursuance to the provisions of this Act and the authority by this Act given to the said State Board of Health, or transgressing the rules, orders and regulations of the said State Board of Health adopted for the removal of the sources of danger from tuberculosis in milch cows or other cattle, or any infectious or contagious disease in domestic animals in any part of the State, shall be guilty of a misdemeanor, and shall also be liable to pay a fine of one hundred dollars, which fine the State Board of Health is hereby authorized to sue for and collect in its name in any court of this State.

SEC 6. All expenses incurred by the State Board of Health in carrying out the provisions of this Act and in performing the duties hereby devolved upon it shall be audited by the Comptroller as other expenses of the said State Board of Health, and the sum of five thousand dollars, or so much thereof as may be necessary, is herewith appropriated out of any money in the treasury not otherwise appropriated for carrying out the provisions of this Act.

SEC. 7. The actual value at the time they are killed, of any animals slaughtered, under the provisions of this law, to be ascertained and determined as hereinafter provided, may be paid to the owners of such cattle under and pursuant to any resolution of the said State Board of Health providing for such payment, and the Board of Claims shall have exclusive jurisdiction to hear, audit and determine all claims which shall arise under the provisions of this Act and to allow thereon such sums as should be paid by the State; provided, however, that no compensation shall be made under the provisions of this Act or otherwise to any person who shall willfully have concealed the existence of disease among his animals, or upon his premises, or who shall in any way directly or indirectly, by act or by willful neglect, have contributed to the spread of disease sought to be suppressed or prevented; and provided, further, that such claims shall not have accrued more than two years prior to the filing of the said claims.

SEC. 8. This Act shall take effect immediately.

